# Clarithromycin impairs tissue-resident memory and Th17 responses to macrolide-resistant *Streptococcus pneumoniae* infections

**DOI:** 10.1007/s00109-021-02039-5

**Published:** 2021-02-17

**Authors:** Marc Lindenberg, Luis Almeida, Ayesha Dhillon-LaBrooy, Ekkehard Siegel, Birgitta Henriques-Normark, Tim Sparwasser

**Affiliations:** 1 Institute of Infection Immunology, TWINCORE, Centre for Experimental and Clinical Infection Research, Hanover, Germany; 2 Institute of Medical Microbiology and Hospital Epidemiology, Hannover Medical School, Hanover, Germany; 3 German Centre for Infection Research, partner site Hanover-Brunswick, Hanover, Germany; 4 Institute of Medical Microbiology and Hygiene, University Medical Center of the Johannes Gutenberg-University, Mainz, Germany; 5 Department of Microbiology, Tumor and Cell Biology, MTC, Karolinska Institutet, Stockholm, Sweden; 6 Clinical Microbiology, Karolinska University Hospital, Stockholm, Sweden

**Keywords:** *Streptococcus pneumoniae*, Macrolide antibiotics, Clarithromycin, Anti-microbial resistance, Tissue-resident memory T cells, Th17 cells

## Abstract

**Abstract:**

The increasing prevalence of antimicrobial resistance in pathogens is a growing public health concern, with the potential to compromise the success of infectious disease treatments in the future. Particularly, the number of infections by macrolide antibiotics-resistant *Streptococcus pneumoniae* is increasing. We show here that Clarithromycin impairs both the frequencies and number of interleukin (IL)-17 producing T helper (Th) 17 cells within the lungs of mice infected with a macrolide-resistant *S. pneumoniae* serotype 15A strain. Subsequently, the tissue-resident memory CD4^+^ T cell (Trm) response to a consecutive *S. pneumoniae* infection was impaired. The number of lung resident IL-17^+^ CD69^+^ Trm was diminished upon Clarithromycin treatment during reinfection. Mechanistically, Clarithromycin attenuated phosphorylation of the p90-S6-kinase as part of the ERK pathway in Th17 cells. Moreover, a strong increase in the mitochondrial-mediated maximal respiratory capacity was observed, while mitochondrial protein translation and mTOR sisgnaling were unimpaired. Therefore, treatment with macrolide antibiotics may favor the spread of antimicrobial-resistant pathogens not only by applying a selection pressure but also by decreasing the natural T cell immune response. Clinical administration of macrolide antibiotics as standard therapy procedure during initial hospitalization should be reconsidered accordingly and possibly be withheld until microbial resistance is determined.

**Key messages:**

• Macrolide-resistant *S. pneumoniae* infection undergoes immunomodulation by Clarithromycin

• Clarithromycin treatment hinders Th17 and tissue-resident memory responses

• Macrolide antibiotics impair Th17 differentiation in vitro by ERK-pathway inhibition

**Supplementary Information:**

The online version contains supplementary material available at 10.1007/s00109-021-02039-5.

## Introduction

Pneumonia represents a major public health concern worldwide, regardless of established antibiotic and supportive treatment regimens. In 2017, 808,902 children under 5 years of age died as a result of pneumonia, with *S. pneumoniae* infections estimated to be the leading cause [1, 2]. In recent years, CD4^+^ tissue-resident memory T cells producing IL-17 (Trm17) were identified as important mediators of neutrophil activation, conferring protection against subsequent *S. pneumoniae* infections [3–5]. Smith et al. showed that secondary protection conferred by a memory T cell response was limited to the lung lobe, which had been already infected once, and was absent in the contralateral one. Moreover, treatment with CD4-depleting or IL-17-neutralizing antibodies impaired this protective response, showcasing the importance of Trm17 cells in secondary infections [4]. Accordingly, in a lung infection model of *Bordetella pertussis*, preventing the egress of lymphocytes from lymphatic organs with the FTY720 antibody during the secondary infection did not impair pathogen clearance [6]. Moreover, parabiosis experiments revealed that tissue-resident T cells rather than migrating cells are the main players in mediating mucosal memory immunity to lung pathogens [7]. Taken together, the studies mentioned above indicate that Trm cells are central in host protection to secondary lung infections. Of particular, importance is their capacity to mediate this protection in an *S. pneumoniae* serotype-independent fashion as currently available vaccines only protect for a certain set of serotypes.

Trm cells are characterized and commonly identified by the permanent expression of CD69. Upon tissue entry, CD69 mediates downregulation of the sphingosine-1-P receptor (S1PR), which promotes the egress of T cells from secondary lymphatic organs into tissues [8]. This reduced expression of S1PR underlines the tissue residency of Trm at the site of possible reactivation in contrast to other T cell memory subsets circulating through secondary lymphatic organs [7, 9]. As the majority of studies focused on CD8^+^ Trm cells, the knowledge of CD4^+^ Trm cells and their respective subclasses is still limited. It is still unclear how the differentiation of this unique cell subset and its reactivation in secondary infections is promoted.

Since pathogen identification is time-consuming and cost-intensive, empirical antibiotic therapy is the method of choice to treat pneumonia in a clinical setting. To account for so-called atypical pathogens like chlamydia, legionella, or mycoplasma species, seriously ill patients can be treated with antibiotic combination regimens that include macrolides like Clarithromycin or Azithromycin [10]. These substances show good antimicrobial capacities against gram-positive and intracellular bacteria. However, in recent years, an increasing frequency of clinical *S. pneumoniae* isolates showed resistance against macrolide antibiotics mediated either by expression of an efflux pump, ribosomal dimethylation, or modification of the ribosomal target site at the S50-subunit [11]. Serotype 15A clinical isolates were increasingly identified to be resistant to multiple antibiotics and to cause invasive pneumococcal disease around the world [12, 13]. Therefore, the likelihood of administering macrolide antibiotics to patients infected with a macrolide-resistant *S. pneumoniae* isolate is rising.

Despite their antibiotic properties, Clarithromycin and Azithromycin can directly modulate host immune cells. Importantly, they have been used successfully to treat inflammatory conditions like diffuse panbronchiolitis, asthma, or cystic fibrosis [14, 15]. Upon macrolide treatment, macrophages displayed an anti-inflammatory phenotype, and dendritic cells showed a lower degree of activation when compared with untreated cells in vitro [16, 17]. Furthermore, Ratzinger et al. showed an inhibitory effect of macrolide antibiotics on human CD4^+^ T helper (Th) 1, Th2, and Th17 cell subsets, which was attributed to mTOR signaling inhibition [18].

While the effects of macrolides on immune cell-mediated inflammation have been thoroughly investigated, there is a lack of research on how the immune response is affected by macrolide treatment during infections by macrolide-resistant pathogens. Here, we sought to investigate the effects of macrolide antibiotics on Th17 cell differentiation, with a special focus on Trm17 cells in the context of macrolide-resistant *S. pneumoniae* infections. We could demonstrate that Clarithromycin treatment impairs not only Th17 differentiation in vitro but also the development of Trm17 responses to a secondary *S. pneumoniae* infection in vivo. Clarithromycin-mediated immunomodulation may thereby favor the spread of resistant isolates by impeding Trm17 immune responses.

## Results

### Clarithromycin treatment impairs T cell response to a macrolide-resistant *S. pneumoniae* isolate

**Fig. 1:**
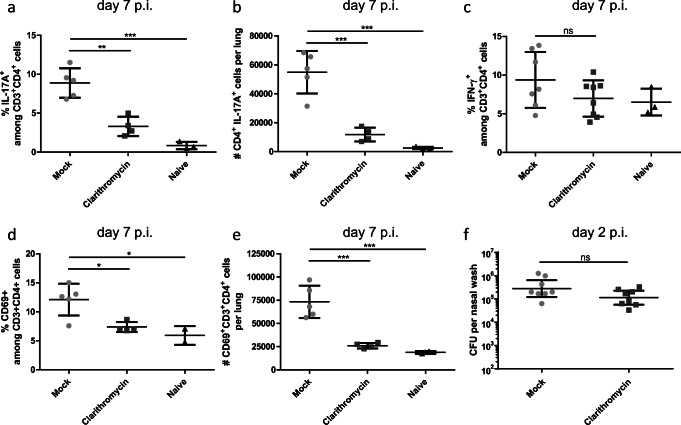
Clarithromycin treatment impairs Th17 response against *S. pneumoniae* infection in vivo. Mice were infected intranasally (i.n.) with 1–1.5 × 10^6^ CFU of macrolide-resistant *S. pneumoniae* clinical isolate (serotype 15A) under ketamine/xylazine anesthesia and treated with 10 mg/kg bodyweight Clarithromycin twice daily for 7 days. **a** Frequencies and **b** total cell numbers of IL-17A^+^ cells among CD4^+^ T cells isolated from the lung at day 7 p.i. and analyzed by FACS. **c** The graph shows frequencies of IFN-γ^+^ T cells. **d** Frequencies and **e** total cell number of CD69^+^ T cells stained directly after isolation from the lungs at day 7 p.i. Data shown are representative of one out of three independent experiments with 3–5 mice per group and depicted as mean ± SD. **f** Bacterial counts of nasal washes performed at day 2 p.i. are pooled from two independent experiments with *n* = 4 mice per group and depicted as geometric mean ± 95% CI. Two-tailed, unpaired Student’s *t* test was used to determine significance between means of groups. ns (not significantly different); *p < 0.05; **p < 0.005; ***p < 0.0005

Since an empirical macrolide antibiotic treatment of patients infected with a macrolide antibiotic-resistant *S. pneumoniae* isolate is likely to occur, we investigated the effects of this treatment on the adaptive T cell response in a mouse model of pneumonia. Therefore, we infected mice with a sub-lethal dose of 1–1.5 × 10^6^ colony forming units (CFU) of a highly macrolide-resistant serotype 15A clinical isolate (MIC 256 μg/ml), which is not included in currently available vaccines. Within the lung tissue of Clarithromycin-treated mice, we found diminished frequencies and numbers of CD4^+^ IL-17A^+^ T cells (Fig. 1a, b) when compared with mock treatment, while the frequency and numbers of CD4^+^ IFN-gamma^+^ T cells (Fig. 1c, S1a) were unimpaired 7 days post-infection (p.i.). Moreover, the frequency and total cell numbers of CD69^+^ CD4^+^ T cells were reduced (Fig. 1d, e), suggesting an overall immunosuppressive capacity of Clarithromycin on T cell activation. To assess the antimicrobial effect of the antibiotic treatment, we investigated the bacterial burden in the lung tissue of mice 7 days p.i. We observed only minimal colony growth, suggesting successful clearance of the infection in both experimental groups (data not shown). To further clarify this aspect, we assessed the bacterial burden in the nasal cavity, which is the main site of bacterial colonization, already on day 2 p.i. However, we did not find any significant difference in the bacterial load as a result of Clarithromycin treatment (Fig. 1f). Hence, we concluded that Clarithromycin treatment dampens the Th17 response without altering the bacterial burden during the early stages of infection.

### Macrolide antibiotics impair Th17 differentiation in vitro

**Fig. 2:**
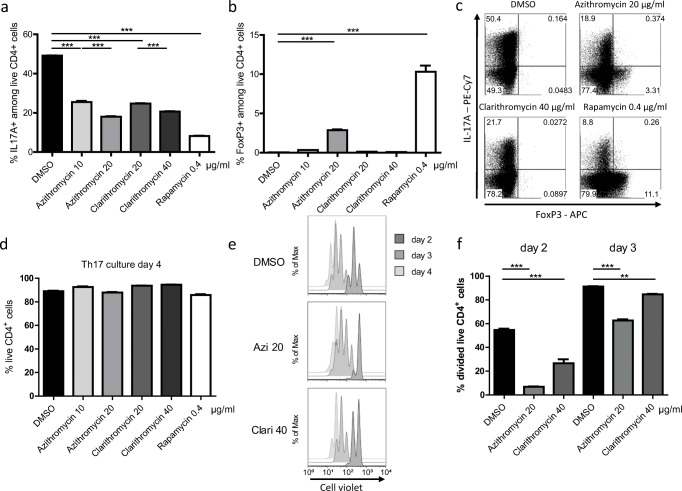
Effects of macrolide antibiotics Azithromycin and Clarithromycin on in vitro Th17 differentiation. Naïve CD4^+^ T cells were isolated from spleen and lymph nodes of mice and isolated by negative magnetic selection. Cultures under Th17 polarizing conditions were performed in 96-well plates, while 100,000 cells per well were seeded and analyzed on day 4. Graphs show **a** percentage of live IL-17A^+^ CD4^+^ T cells and **b** FoxP3^+^ T cells determined by **c** FACS analysis. **d** Cell viability is depicted as frequency of live CD4^+^ T cells. **e** Histogram showing CD4^+^ T cells stained with CellViolet™ to visualize proliferation at different time points. **f** Frequencies of divided CD4^+^ T cells upon indicated treatments at days 2 and 3 of the Th17 culture. Data shown are representative of at least three independent experiments, each performed in technical triplicates and depicted as mean ± S.E.M. Two-tailed, unpaired Student’s *t* test was used to determine significance between means of groups. **p < 0.005; ***p < 0.0005

A variety of host cells were shown to be affected by macrolide antibiotics [16–18]. To test if the in vivo phenotype could be explained by a direct effect of macrolides on Th17 cells, we cultured murine naïve CD4^+^ T cells under Th17 polarizing conditions in the presence of Clarithromycin, Azithromycin, or the immunosuppressive drug Rapamycin as a control. We found an apparent reduction in the frequency of IL-17A^+^ cells for all drug treatments (Fig. 2a). The expression of hallmark transcription factor retinoic acid receptor-related orphan receptor gamma t (ROR gamma t) was unaffected, and already on day 2 of culture, nearly all live CD4^+^ T cells stained positive (Fig. S2a). Unlike Rapamycin, neither Azithromycin nor Clarithromycin led to the expression of FoxP3 in a significant proportion of cells by the end of the Th17 culture (Fig. 2b, c). To check further for effects on regulatory T cell (Treg) differentiation, we performed inducible Treg (iTreg) cultures. Only a slight reduction upon treatment with Azithromycin could be observed (Fig. S2b). Cell viability was not impaired upon treatment with the indicated macrolide concentrations in Th17 cultures (Fig. 2d); however, in iTreg cultures, viability was reduced in the higher dosages tested. Moreover, we could not detect an influence of the macrolide antibiotics on Th1 differentiation, assessed by IFN-gamma production, or expression of hallmark transcription factor T-bet (Fig. S2c, S2d). As proliferation and cytokine production of T cells are intimately linked, we aimed to determine if the macrolide-mediated reduction in cytokine expression was a result of impaired cellular proliferation. By labeling cells with CellTrace™ dye prior to the Th17 culture, we were able to observe that proliferation was strongly inhibited on the second day but only mildly impaired at later time points when compared with untreated cells (Fig. 2e, f). Rapamycin treatment resulted in a strong reduction in proliferation by day 4, which was more attenuated than Clarithromycin (Fig. S2e). In vitro macrolide treatment, therefore, inhibits Th17 differentiation and negatively affects early activation and proliferation events.

### Macrolide antibiotics treatment hinders ERK-pathway activation in Th17 cells

**Fig. 3:**
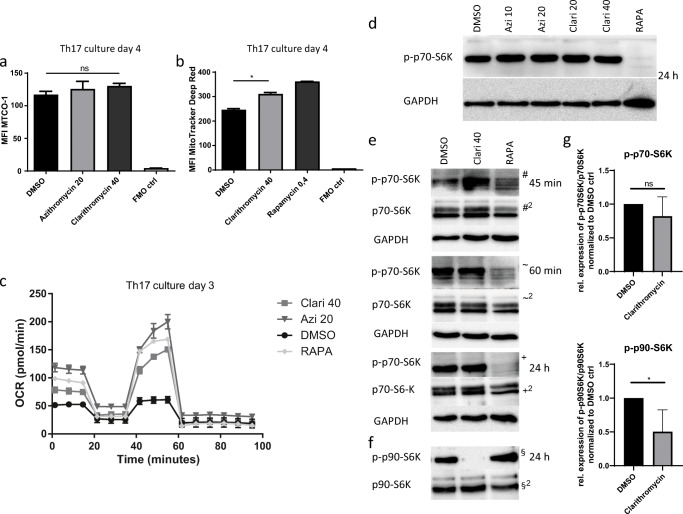
Analysis of mitochondrial capacity and signaling pathways of macrolide antibiotic-treated in vitro differentiated Th17 cells. **a** FACS analysis of mitochondrial cytochrome c oxidase depicted as mean fluorescent intensity (MFI) at day 4 of culture, data shown are representative of one out of two independent experiments, each performed in technical triplicates and depicted as mean ± S.E.M. **b** MFI of MitoTracker Deep Red™ at day 4 of Th17 culture, data shown are representative of one out of three independent experiments, each performed in technical duplicates and depicted as mean ± S.E.M. **c** Oxygen consumption rate of cells at day 3 of Th17 culture in Mito-stress test with subsequent injections of oligomycin, FCCP, rotenone, and antimycin A at the indicated time points. Data from one out of two experiments, each performed in technical triplicates and depicted as mean ± S.E.M. **d** Western blot of cell lysates to identify phosphorylated and unphosphorylated p70-S6-kinase at 24 h of culture, **e** at 45 and 60 min. Membranes were subsequently probed twice with antibodies to visualize phosphorylated and total protein as indicated with a sign plus as superscript 2. **f** Western blot to identify p90-S6-kinase 24 h after start of culture. **g** Quantification of p-p70-S6/p70-S6 and p-p90-S6/p90-S6 of three independent Western blots from independent cultures at 24 h normalized to the respective ratios in DMSO controls. All Western blots for indicated time points were run at least twice and verified initial findings. Two-tailed, unpaired Student’s *t* test was used to determine significance between means of groups. ns (not significantly different); *p < 0.05

Macrolide antibiotics have been documented to affect mitochondrial fitness in different cell lines in vitro [19, 20]. As the mechanistic target of macrolide antibiotics is the S50-subunit of bacterial ribosomes [14], and mitochondria are believed to be of bacterial origin [21], we hypothesized that inhibition of mitochondrial translation could be an effect of macrolide treatment. Recently, metabolic modulation has been shown to influence T cell fate decisions, and the antibiotic linezolid inhibits Th17 cell formation by interfering with mitochondrial translation [22]. However, when we checked for expression of the exclusively mitochondrial translated cytochrome c oxidase, no difference between treated and untreated cultures could be detected (Fig. 3a). Evaluating the mitochondrial mass per cell by MitoTracker Deep Red™ staining, we identified higher mean fluorescence intensities (MFI) in Clarithromycin- and Rapamycin-treated Th17 (Fig. 3b) and Th1 (Fig. S3a) cultures. In addition, mitochondrial membrane potential-dependent staining by tetramethylrhodamine, ethyl ester (TMRE) revealed increased MFI in treated cells compared with DMSO-treated controls in Th17, Th1, and iTreg cultures (Fig. S3b). Furthermore, we investigated the influence of Clarithromycin and Azithromycin treatment on the mitochondrial respiratory capacity of differentiating Th17 cells through a mitochondrial stress assay. We found an unexpected increase in the maximal respiratory rate of macrolide-treated cells and no impairment of mitochondrial respiration (Fig. 3c). The mammalian target of Rapamycin (mTOR) is a central mediator of metabolic changes in effector T cells. Since a direct inhibitory effect on mTOR by Azithromycin has been previously described in human CD4^+^ T cells [18], we tested phosphorylation of the downstream signaling protein p70-S6-kinase (p70-S6K) at day 1 of the culture. While Rapamycin completely abrogated the p-p70-S6K signal as expected, no effects were visible for Azithromycin or Clarithromycin-treated cells compared with control cells (Fig. 3d). As Ratzinger et al. found differences in the phosphorylation of the ribosomal protein S6 (RP-S6) early after human CD4^+^ T cell activation [18], we checked for p70-S6K activation in naïve murine CD4^+^ T cells under TCR activation for 45 min, 60 min, and 24 h in the presence of 40 μg/ml Clarithromycin. No differences between treated and control cells were observed, while Rapamycin showed a strong decrease in phosphorylated p70-S6K levels (Fig. 3e). RP-S6 phosphorylation is not selectively mTOR dependent and mediated by p70-S6K [23], but can be facilitated by p44/42-Erk-kinase (Erk1/2) activation leading to subsequent p90-ribosomal-S6-kinase (p90-S6K; RSK) activation. As macrolides are described to interfere with Erk1/2 signaling [24], we analyzed p90-S6K phosphorylation. Interestingly, we found a significant reduction of the phosphorylated p90-S6K protein to total protein ratio in Clarithromycin-treated cells at day 1, while Rapamycin showed no effect (Fig. 3f, g). Moreover, analysis of Erk1/2 phosphorylation showed a modest increase in Clarithromycin und Rapamycin-treated cells suggestive of reduced negative feedback loops (Fig. S3c, d). Additionally, we checked for the phosphorylated signal transducers and activators of transcription protein 3 (STAT-3) and found a slight reduction upon Clarithromycin treatment 24 h after the start of culture (Fig. S3e, f). Therefore, Clarithromycin blocks the metabolic changes needed to acquire a mitochondrial effector cell profile and inhibits the Erk1/2 kinase pathway rather than the mTOR pathway in murine Th17 cell differentiation.

### Clarithromycin is not influencing the Trm17 response when treatment only occurred during the first infection

**Fig. 4:**
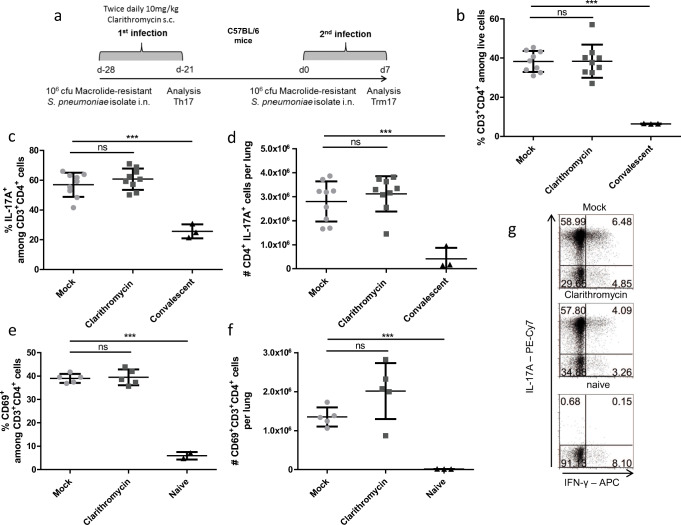
Clarithromycin treatment only during the first infection fails to influence Trm response to the second infection. **a** Graphical visualization of the experimental layout is depicted. **b** Frequencies of CD4^+^ T cells isolated from the lungs are shown at day 7 p.i. **c** Frequencies and **d** total cell numbers of CD4^+^CD3^+^ T cells producing IL17A upon PMA/Ionomycin restimulation are depicted. Shown are **e** frequencies and **f** total cell numbers of CD4^+^ T cells staining positive for CD69. **g** Representative FACS plots visualizing cytokine staining after restimulation are depicted. Data shown represent two pooled independent experiments out of three for **b**, **c**, and **d** (while the control group is only representative for one) and representative of one out of two for **e** and **f** with 5-3 mice per group, and depicted as mean ± S.D. Two-tailed, unpaired Student’s *t* test was used to determine significance between means of groups. ns (not significantly different); ***p < 0.0005

Trm17 cells are crucial in *S. pneumoniae* infections to confer protection against secondary infection in a serotype-independent fashion [3]. Since we found a diminished Th17 response to the first *S. pneumoniae* infection upon Clarithromycin treatment in mice, we investigated the effects on Trm17 cells in a reinfection model. During the initial infection period, we treated the mice with Clarithromycin and waited 3 weeks for the primary infection to clear. After this period, we reintroduced a secondary infection without Clarithromycin treatment (Fig. 4a). Convalescent mice were analyzed as controls to check for potential immune activation still present from the first infection. We found a strong increase in both frequencies among CD4^+^ T cells and total numbers of IL-17A^+^ CD4^+^ T cells upon secondary infection compared with the initial immune response (Fig. 1a, b vs. Fig. 4c, d). Trm cells were essentially committed to IL-17A production and expanded rapidly. These cells were not confounded by cells from the blood circulation at the time point of analysis, as the intravenous application of an antibody against the pan-lymphocyte marker CD45.2 10 min prior to asphyxiation stained only a negligible proportion of CD4^+^ IL-17A^+^ T cells (Fig. S4a). However, Clarithromycin treatment only during the first infection failed to influence the Trm response to the reinfection. The overall frequency of CD4^+^ T cells was unchanged (Fig. 4b), and comparable frequencies and total cell numbers of IL-17A^+^ CD4^+^ T cells were found in the experimental mouse groups (Fig. 4c, d). Moreover, we analyzed the frequencies of CD4^+^ CD69^+^ T cells, but no differences were induced upon Clarithromycin treatment (Fig. 4e, f). Additionally, IFN-gamma producing CD4^+^ T cells increased rather than decreased in Clarithromycin-treated mice compared with mock treatment, while their overall numbers remained low compared with Trm17 cells (Fig. 4g, S4b, c). In line with these findings, both mock- and Clarithromycin-treated groups recovered equally from initial weight loss upon reinfection (Fig. S5a). Hence, although Clarithromycin treatment impairs the initial Th17 response, the Trm17 response upon reinfection in the absence of Clarithromycin was unimpaired.

### IL-17 producing Trm cells in the lung are reduced by Clarithromycin treatment during the first and second infection

**Fig. 5:**
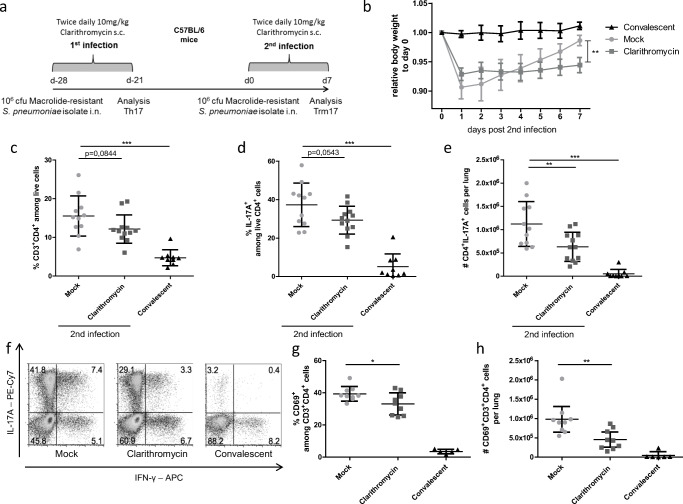
Clarithromycin treatment during both infections attenuated Trm17 response**. a** Graphical visualization of the experimental layout is depicted. **b** Bodyweight curve of mice recovering from the second infection is shown as pooled data from 4 experiments. **c** Frequencies of CD4^+^ T cells isolated from the lungs are shown at day 7 p.i. **d** Frequencies and **e** total cell numbers of CD4^+^CD3^+^ T cells producing IL17A upon PMA/ionomycin restimulation are depicted. **f** Representative FACS plots visualizing cytokine staining after restimulation are depicted. Shown are **g** frequencies and **h** total cell numbers of CD69^+^CD4^+^ T. Data shown are three pooled independent experiments out of three for **b–e** and two pooled out of two for **g** and **h** with 3–5 mice per group and depicted as mean ± S.D. Two-tailed, unpaired Student’s *t* test was used to determine significance between means of groups. ns (not significantly different); *p < 0.05; **p < 0.005; ***p < 0.0005 or as indicated

To complete our study on the effects of Clarithromycin on the Trm17 response to *S. pneumoniae*, we further applied antibiotic treatment during both infection phases (Fig. 5a). We observed a delayed recovery of body weight from the initial loss upon the second intranasal infection. Seven days p.i., Clarithromycin-treated mice weighed considerably less than the mock-treated control group, hinting to a prolonged disease state (Fig. 5b). Hence, we checked for the memory immune cell response in the lung. Indeed, while there was an overall decreased tendency of CD4^+^ T cell frequency and IL-17A^+^ CD4^+^ T cells (Fig. 5c, d), analyzing the total cell number of IL-17A^+^ CD4^+^ T cells from the lung tissue revealed a significant decrease in Clarithromycin-treated mice compared with the mock-treated group (Fig. 5e). No effect could be observed with regard to IFN-gamma producing CD4^+^ T cells (Fig. S5b). IL-17A production correlated with the expression of Th17 transcription factor ROR gamma t upon the first infection as well as upon the second challenge, while overall frequencies were around 20% higher compared with IL17A^+^ CD4^+^ T (Fig. S5c). Moreover, the frequency and number of CD69^+^ CD4^+^ T cells were reduced under Clarithromycin treatment (Fig. 5g, h). Mice, which have been infected once but not reinfected, served as a control group for the secondary infection and exhibited reduced Trm17 responses in terms of IL-17A production and overall CD4^+^ T cell frequencies (Fig. 5 c–h). As we observed an impaired Trm17 response at 7 days p.i., we checked for the bacterial burden in lung homogenates, but clearance already diminished counts below our detection limit of 10 CFU/ml lung homogenate. We concluded that Clarithromycin treatment also impairs the Trm17 response to a consecutive infection, irrespective of its effects on the initial Th17 response.

## Discussion

Macrolide antibiotics like Clarithromycin and Azithromycin are known for their immunomodulatory capacities and have been used to dampen inflammatory conditions [25–28]. Due to time-consuming diagnostics, clinical guidelines still recommend macrolide antibiotic combinations for calculated therapy of patients at risk to cover for atypical pathogens [10]. As we are confronted with increasing antimicrobial resistance rates, the underlying hypothesis of this study was to identify if treatment with Clarithromycin may favor the spread of resistant isolates by impairing the formation of Trm17 cells. These cells have been shown to be of key importance to confer protection against consecutive infections and to do so in a serotype-independent manner [3, 4]. Concerning the development of vaccines, the induction of these cells, therefore, seems to be a good strategy to consider not only selected serotypes but rather *S. pneumoniae* in a serotype-independent fashion. Hence, impairment of Trm17 induction, proliferation, or function must be considered negative for host pathogen defense. We found that Clarithromycin treatment impairs the initial as well as the memory Th17 response to a highly resistant *S. pneumoniae* clinical isolate. While studies in other infection models showed similar effects [29–31], here we used a macrolide-resistant isolate to reduce the bias of antigen reduction. In a study design in which both antimicrobial and immunomodulatory properties of the substance under investigation are at play, the effects of either one on T cells are mechanistically difficult to discriminate. Antigen reduction and therefore decreased levels of inflammatory pathogen-associated molecular patterns (PAMPs) and stimuli are inextricably linked to adaptive T cell immunity. This might mask additional immunomodulatory effects, especially by antibiotics. Recently, Borkner et al. found that Azithromycin hinders Trm cell responses in a murine model of vaccination and subsequent *Bordetella pertussis* infection [31]. Azithromycin treatment during a vaccination phase with inactivated bacteria led to decreased Trm17 responses to subsequent infections with vital bacteria regarding frequency and function. In case of this study, a vaccination phase was used to exclude antigen loss due to antimicrobial activity, as an already inactivated whole pathogen formulation was used for the initial antigen delivery. Interestingly, we found Clarithromycin treatment to be necessary during the second infection to detect a reduced Trm17 cell response. In our consecutive infection model, we could not detect altered Trm17 numbers, when we applied treatment only during the first infection, even though the primary response was impaired. As a real infection is hardly comparable with vaccinations regarding induction of tissue-resident memory cells, further studies are necessary to elucidate how induction and reactivation of CD4^+^ Trm cells are regulated and which threshold exists to implement a sufficient memory response. One interesting subset to be analyzed with respect to their influence on Trm responses are Tregs, as known regulators for effector T cells. Also, the analysis of tumor necrosis factor (TNF)-alpha would extend the analysis of Trm cells especially with regard to limiting invasive pneumococcal infections [32].

Many different immune cell subsets besides Th cells have been described to be influenced by macrolide antibiotics, which accumulate intracellularly [33–36]. Due to this accumulation, we used a dose of 10 mg/kg body weight twice daily for our in vivo experiments to further minimize antigen reducing effects. Higher doses in mice mimicked the area under the curve (AUC) in 24-h measurements in clinical patients with 500 mg Clarithromycin twice daily [37], but studies described effects on the expression of pathogenicity factors of resistant *S. pneumoniae* even if the bacterial load was unaffected [38]. We, therefore, sought to keep the antimicrobial influence as low as possible. The concentrations used in vitro corresponded approximately to tissue levels measured in pharmacological studies [39].

Ratzinger et al. reported on the inhibition of human Th1, Th2, and Th17 cells in vitro with identical macrolide concentrations, also used in this work [18]. To elucidate the underlying mechanism, they checked for RP-S6 activation as a readout for mTOR activity and found reduced phosphorylation levels compared with control cells. Differences were found at 24 h by FACS analysis, and 45 and 60 min after T cell stimulation through Western blot quantification. While we found inhibitory effects in murine Th17 cultures, we failed to detect differences in p70-S6K phosphorylation at these time points. p70-S6K is placed directly downstream of mTOR and phosphorylates RP-S6. Hence, inhibition of mTOR should directly influence its activation. Moreover, while Rapamycin, as a selective mTOR inhibitor, induced regulatory T cells under Th17 polarizing conditions, as shown before [40], Clarithromycin failed to do so. This led us to investigate other pathways resulting in RP-S6 phosphorylation since RP-S6 is not an exclusive mTOR target. Previous results in other cell types pointed towards an ERK-pathway inhibition by Clarithromycin and Azithromycin [41]. ERK activation also leads to phosphorylation of RP-S6 via the p90-S6K. In addition, we found a clear reduction in p90-S6K phosphorylation at 24 h of culture. Moreover, inhibition of this kinase was described to result in enhanced phosphorylation of ERK itself, as multiple negative feedback loops are described to regulate the ERK pathway [42, 43]. Downstream inhibition of the ERK pathway by Clarithromycin is therefore likely to result in the modest increase in phosphorylation of the ERK protein we observed. ERK pathway inhibition was shown to impair glucose uptake and the upregulation of glycolysis in activated T cells [44]. Naïve T cells undergo a metabolic shift to meet increased energy and biosynthetic demands upon activation. A disturbed activation of glycolysis seems to result in an increase in mitochondrial respiration as an inevitable consequence, as previously described for two ERK pathway inhibitors in two cancer cell lines [45]. Hence, in our experimental setting, the downstream activation of the ERK rather than the mTOR pathway is affected and mediates macrolide inhibition of Th17 cell formation. If these discrepant observations recapitulate species-specific differences remains to be elucidated.

During macrolide antibiotic therapy of pneumonia, monotherapy is very unlikely to happen in the clinics. Therefore, the influence of macrolide antibiotics on the immune response seems to be negligible as the other antibiotics will reduce the antigen load. However, many clinical isolates harbor not only one but multiple resistances to other antibiotics. The isolate we used was also confirmed to be resistant against Clindamycin (MIC 256 μg/ml) and Tetracycline (MIC 64 μg/ml), and others already showed high levels of penicillin resistance. Moreover, in light of declining development rates of new antimicrobial substances and increasing frequencies of multi-resistant bacterial strains, the prevention of infection and early control is of vital importance. Thus, Trm cells and vaccination strategies inducing those cells are highly warranted as they are perfectly situated to react fast in a serotype-independent fashion. An ineffective therapy that also impairs Trm cell function should, therefore, be avoided. The direct translation of murine data into human treatment recommendations is not possible, but studies investigating antibiotic effects on Trm formation in patients are urgently needed, although access to tissue material is likely to be the limiting factor. Nevertheless, the important role of Trm cells central to the lung memory response is well established in human studies [46]. The anticipation of rising antimicrobial resistance rates in the near future demands critical evaluation of antibiotics for their immunomodulatory capacities.

## Material and methods

### Mice and infections

Male C57BL6 mice aged between 8 and 12 weeks at the time point of the first infection were infected once or twice with an intranasal inoculum containing 1–1.5×10^6^ CFU *S. pneumoniae* isolate 89/17 tested for a high macrolide resistance (MIC 256 μg/ml) under ketamine/xylazine anesthesia. *S. pneumoniae* was grown, as recently described [47], and CFU contained in the inoculum was determined as described before [48]. To allow the inoculum to reach the lungs, mice were kept in a vertical position for around 1 min. A monitoring phase of 1 week after each infection and a 3-week time frame between infections was conducted. Inoculation dosage was checked by plating inoculum on blood agar plates in serial dilutions after administration. Mice were treated subcutaneously with 10 mg/kg Clarithromycin (Cayman Chemicals) twice daily, and controls were injected with the carrier solution of 1:10 EtOH in 0.9% NaCl only. Mice were bred and maintained under specific pathogen-free conditions at the animal facility at TWINCORE (Hannover, Germany). All animal experiments were performed in compliance with the German animal protection law (TierSchG BGBl. I S. 1105; 25.05.1998) and were approved by the Lower Saxony Committee on the Ethics of Animal Experiments as well as the responsible state office (Lower Saxony State Office of Consumer Protection and Food Safety) under the permit number 33.19-42502-04-17/2688.

### Bacterial counts

Nasal washes were performed by exposing the trachea and inserting a cannula retrograde to flush the nasal cavity with ice-cold PBS. The first ten drops were collected from the nostrils and plated in serial dilution on blood agar plates. The next day, colonies were counted, and CFU per nasal wash were determined. CFU in the lungs were determined by serial dilution of tissue homogenates.

### In vitro cultures

Naïve CD4^+^ T cells were isolated from spleens and lymph nodes of mice by enrichment with EasyStep® Mouse CD4^+^ Isolation Kit (Stemcell Technologies). Incubation time for the provided antibody mix was 10 min while additionally, anti-CD25-biotin (1 μg/ml final concentration, Invitrogen) and anti-CD44-biotin (0.5 μg/ml final concentration, Invitrogen) were added for the last 2.5 min followed by magnetic negative isolation with streptavidin beads as described by the manufacturer. A purity of more than 90% of naïve T cells (determined by CD69, C62L, CD25, and CD44 expression) was achieved. IMDM GlutaMAX® medium (Life Technologies) supplemented with 10% heat-inactivated FCS (Biochrom), 500 U penicillin-streptomycin (PAA laboratories), and 50 μM β-mercaptoethanol (Life Technologies) and 96-well flat bottom plates were used for cultivation. For Th17 induction, 1.0 × 10^5^ naïve T cells were seeded and cultured for 4 days with plate-bound αCD3 (10 μg/ml, clone 145-2C11; Bio X Cell), αCD28 (1 μg/ml, clone 37.51; Bio X Cell), αIFN-gamma (5 μg/ml, clone XMG1.2; Bio X Cell), αIL-4 (5 μg/ml, clone 11B11; Bio X Cell), rhTGF-β1 (2 ng/ml; Peprotech), rmIL-6 (10 ng/ml; Peprotech), and rmIL-1β (50 ng/ml; Peprotech). For iTreg induction, 5.0 × 10^4^ naïve T cells were seeded and cultured for 4 days with plate-bound αCD3 (10 μg/ml, clone 145-2C11; Bio X Cell), αCD28 (1 μg/ml, clone 37.51; Bio X Cell), rhTGF-β1 (3 ng/ml; Peprotech), and rhIL-2 (200 U/ml; Roche). For Th1 induction, 1.0 × 10^5^ naïve T cells were seeded and cultured for 4 days with plate-bound αCD3 (10 μg/ml, clone 145-2C11; Bio X Cell), αCD28 (1 μg/ml, clone 37.51; Bio X Cell), αIL-4 (10 μg/ml, clone 11B11; Bio X Cell), and rmIL-12 (20 ng/ml; Peprotech).

### FACS staining

The following conjugated monoclonal antibodies from eBioscience/ThermoFisher were used: CD3 APC-efluor780 (17A2), CD4 efluor450 (RM4-5), CD45.2 APC (104), CD62L PE-Cy7 (MEL-14), CD69 PE (H1.2F3), Foxp3 APC (FJK-16s), IL-17A PE-Cy7 (eBio17B7), IFN-gamma efluor660 (XMG1.2), ROR-gamma-t PE (B2D), and T-bet efluor660 (eBio4B10). Dead cells were excluded by LIVE/DEAD® Fixable Dead Cell Stain Kit (Life Technologies). For intracellular cytokine staining, cells were stimulated with Phorbol 12-myristate 13-acetate (0.1 μg/ml/1; Sigma-Aldrich) and ionomycin (1 μg/ml/1; Sigma-Aldrich) for 4h, the last 2h in the presence of Brefeldin A (5 μg/ml), stained for surface markers, fixed using Foxp3/Transcription Factor Fixation/Permeabilization Kit (Affymetrix/eBioscience) according to manufacturer’s instruction, and stained with the respective antibodies against cytokines or transcription factors diluted in PBS containing 0.25% BSA and 0.5% of Saponin. The acquisition was performed on an LSR II flow cytometer (Becton Dickinson), and data were analyzed with FlowJo software (Tree Star, Inc.). Single stains were used for compensation and fluorescence minus one (FMO) controls for gating. For the MT-CO1 (Abcam, EPR19628), staining cells were fixed with ice-cold methanol.

### Western blot

Whole-cell lysates were prepared at the indicated time points after the start of culture using lysis buffer (Pierce™ RIPA buffer, Thermo Scientific) supplemented with 10mM natriumfluorid, phenylmethylsulfonyl fluoride (PMSF), and CLAP (chymostatin, leupeptin, antipain, and pepstatin) to inhibit dephosphorylation and degradation. Cell lysates were separated by SDS-gel electrophoresis and transferred to PVDF membranes (Merck Millipore). Anti-p-p70-S6-kinase (Thr389; 108D2), anti-p70-S6-kinase (49D7), anti-p-p90-S6-kinase (p-p90RSK; Thr573), anti-p90-S6-kinase (RSK1/2/3; 32D7, anti-p-p44/42 MAPK (Erk1/2; Thr202/Tyr204; D13.14.4E), anti-p44/42 MAPK (Erk1/2; 137F5), anti-pSTAT3 (Tyr705; D3A7), and GAPDH (D16H11) were used for immunoblotting and goat-anti-rabbit horseradish peroxidase (HRP) (all from Cell Signaling) and pico-ECL or femto-ECL (Thermo Fisher) according to signal strength for detection. Anti-beta-actin (AC-15) was received from Sigma and goat-anti-mouse HRP from Jackson ImmunoResearch Lab Inc. Immunoblotting of phosphorylated proteins was followed after washing with antibodies against the unphosphorylated protein to compare expression. Blots of the same membrane incubated with antibodies for a second time are indicated with *a*^2^ in superscript. Semiquantitative analysis was performed using ImageJ (Fiji), and the signal strength of phosphorylated to total protein was compared. GAPDH or beta-actin served as a loading control.

### Analysis of mitochondrial capacity

In vitro Th17-differentiated cells were harvested after 96 h of culture and plated on 96-well XF cell culture microplates in XF assay medium (pH 7.4, Agilent) supplemented with D-glucose (10 mM) and L-glutamine (4 mM, Gibco) with a density of 3 × 10^5^ cells per well. Cells were incubated for 30 min at 37 °C in a non-CO_2_ incubator, and the oxygen consumption rate OCR was analyzed using an XF96 Extracellular Flux Analyzer (Agilent). For the mitochondrial stress assay analysis, the XF Mitochondrial Stress Test was performed as recommended by the manufacturer, using subsequent injections of oligomycin (1 μM), FCCP (1 μM), rotenone (10 μM), and antimycin A (10 μM). Mitochondrial staining was performed by the use of MitoTracker Deep Red™ (ThermoFisher Scientific) at a final concentration of 250 nM in supplemented IMDM GlutaMax medium for 30 min at 37°C. The mean fluorescent intensity was analyzed after additional staining with LIVE/DEAD™ Fixable Dead Cell Stain Kit (Life Technologies) to gate on live cells. Additionally, staining with TMRE at a final concentration of 400 nM was performed with the same parameters, while the staining control was incubated with FCCP for 10 min prior to incubation with TMRE.

### Statistics

Data analysis was performed using GraphPad Prism Software 6.0. Statistics were calculated using Student’s *t* test. Means are given as ± S.D. or where indicated as ± S.E.M., with *P* values considered significant as follows: *p < 0.05, **p < 0.005, and ***p < 0.0005.

## Supplementary information

Suppl. Fig. 1 Total cell numbers of IFN gamma producing CD4^+^ T cells. **a** Total cell numbers of IFN gamma producing CD4^+^ T cells upon PMA/ionomycin ex vivo restimulation at day 7 p.i. Data shown are two pooled experiments with 3–5 mice per group, and depicted as mean ± S.D. Two-tailed, unpaired Student’s *t* test was used to determine significance between means of groups. ns (not significantly different) (PNG 1590 kb)

High resolution image (EPS 590 kb)

Suppl. Fig. 2 In vitro effects of macrolide antibiotics on different Th subset differentiation and Th17 proliferation. **a** ROR gamma t expression in CD4^+^ T cells treated with the indicated macrolide concentrations at day 2 of Th17 culture. **b** Frequencies of FoxP3 expressing cells at day 4 of iTreg culture. **c** Frequencies of T-bet expressing cells and **d** IFN gamma producing CD4^+^ T cells at day 4 of Th1 culture. **e** Cell violet™ staining of Th17 differentiated CD4^+^ T cells at day 4 of culture. Data shown are representative of at least two individual experiments with three technical replicates each and depicted as mean ± S.E.M. Two-tailed, unpaired Student’s *t* test was used to determine significance between means of groups. ns (not significantly different); *p < 0.05 (PNG 4469 kb)

High resolution image (EPS 1790 kb)

Suppl. Fig. 3 In vitro effects of macrolide antibiotics on mitochondrial staining, ERK, and STAT3 phosphorylation. **a** Mitochondrial staining with MitoTracker Deep Red™ depicted as mean fluorescence intensity (MFI) at day 4 of Th17 culture. **b** Mitochondrial membrane potential-dependent staining with tetramethylrhodamine ethyl ester (TMRE) depicted as mean fluorescence intensity (MFI) at day 4 of Th17, Th1, and iTreg culture. Control cells were treated with FCCP to impair the mitochondrial membrane potential and thereby TMRE staining. Data shown are representative of three individual experiments with two technical replicates and depicted as mean ± S.E.M. Two-tailed, unpaired Student’s *t* test was used to determine significance between means of groups. ns (not significantly different); ***p < 0.0005. **c** Representative Western blots of p-ERK1/2 and ERK1/2 proteins of cells treated with 40 μg/ml Clarithromycin, 0.4 μg/ml Rapamycin, or DMSO as control and harvested at 24 h of Th17 culture. **d** Quantification of the expression of p-ERK1/2 to the total ERK1/2 expression normalized to this ratio in the DMSO control. **e** Representative Western blots of p-STAT3 and beta actin of cells treated with 40 μg/ml Clarithromycin, 0.4 μg/ml Rapamycin, or DMSO as control and harvested at 24 h or day 4 of Th17 culture. **f** Quantification of the expression of p-STAT3 to beta actin expression normalized to this ratio in the DMSO control at 24 h. Data shown are semi-quantitative analysis of two independent blots without further statistical analysis (PNG 4571 kb)

High resolution image (EPS 1932 kb)

Suppl. Fig. 4 Analysis of the memory Trm1 response and confirmation of tissue-residency of Trm17 cells. **a** Representative FACS plots of lung cells isolated from C57BL6 mice that have been infected with consecutive *S. pneumoniae* infections and intravenous application of a fluorescent conjugated antibody for the lymphocyte marker CD45.2 10 min before asphyxiation to discriminate blood circulating and lung resident cells. On the right-hand side, IL-17A staining showed only a negligible fraction of APC positive cells producing IL-17A, and on the left-hand side, CD69^+^ cells were not confounded by blood circulating cells, when stained directly ex vivo without restimulation. **b** Frequencies and **c** total cell numbers of IFN gamma producing CD4^+^ T cells at day 7 of the second *S. pneumoniae* infection. Data shown are two pooled independent experiments out of three (while the control group is only representative for one), and depicted as mean ± S.D. Two-tailed, unpaired Student’s *t* test was used to determine significance between means of groups. ns (not significantly different); *p < 0.05 (PNG 5706 kb)

High resolution image (EPS 1800 kb)

Suppl. Fig. 5 Bodyweight curve of mice recovering from the second infection without Clarithromycin treatment; analysis of the memory Trm1 response and correlation of ROR gamma t expression to IL-17A production of Trm17 cells upon Clarithromycin treatment. **a** Bodyweight curve of mice infected for the second time with *S. pneumoniae* only treated with Clarithromycin or mock during the first infection. Data are pooled from three independent experiments. **b** Frequencies and total cell numbers of IFN gamma producing CD4^+^ T cells at day 7 of the second *S. pneumoniae* infection, while Clarithromycin treatment occurred during both infections. Data shown are two pooled independent experiments out of three, and depicted as mean ± S.D. **c** Frequencies of ROR gamma t expressing (left panel) and IL-17A producing CD4^+^ T cells from one experiment out of two, depicted as mean ± S.D. Two-tailed, unpaired Student’s *t* test was used to determine significance between means of groups. ns (not significantly different), *p* value as indicated (PNG 4128 kb)

High resolution image (EPS 1444 kb)

## Data Availability

Raw data and material of the study are available upon request from the corresponding author.
