# Supplementary figures and images for: Clarithromycin impairs tissue-resident memory and Th17 responses to macrolide-resistant Streptococcus pneumoniae infections
Source: J Mol Med (Berl). 2021 Feb 17;99(6):817–29. doi: 10.1007/s00109-021-02039-5 (PMC8164591; doi:10.1007/s00109-021-02039-5)

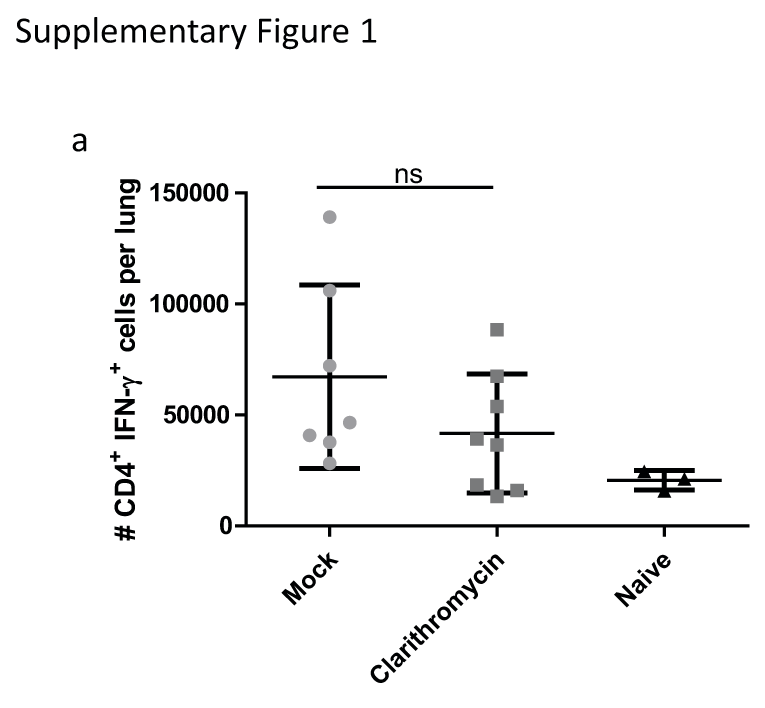

Supplement: Supplementary file 1 — Total cell numbers of IFN gamma producing CD4+ T cells. a Total cell numbers of IFN gamma producing CD4+ T cells upon PMA/ionomycin ex vivo restimulation at day 7 p.i. Data shown are two pooled experiments with 3–5 mice per group, and depicted as mean ± S.D. Two-tailed, unpaired Student’s t test was used to determine significance between means of groups. ns (not significantly different) (PNG 1590 kb) [file 109_2021_2039_Fig6_ESM.png]

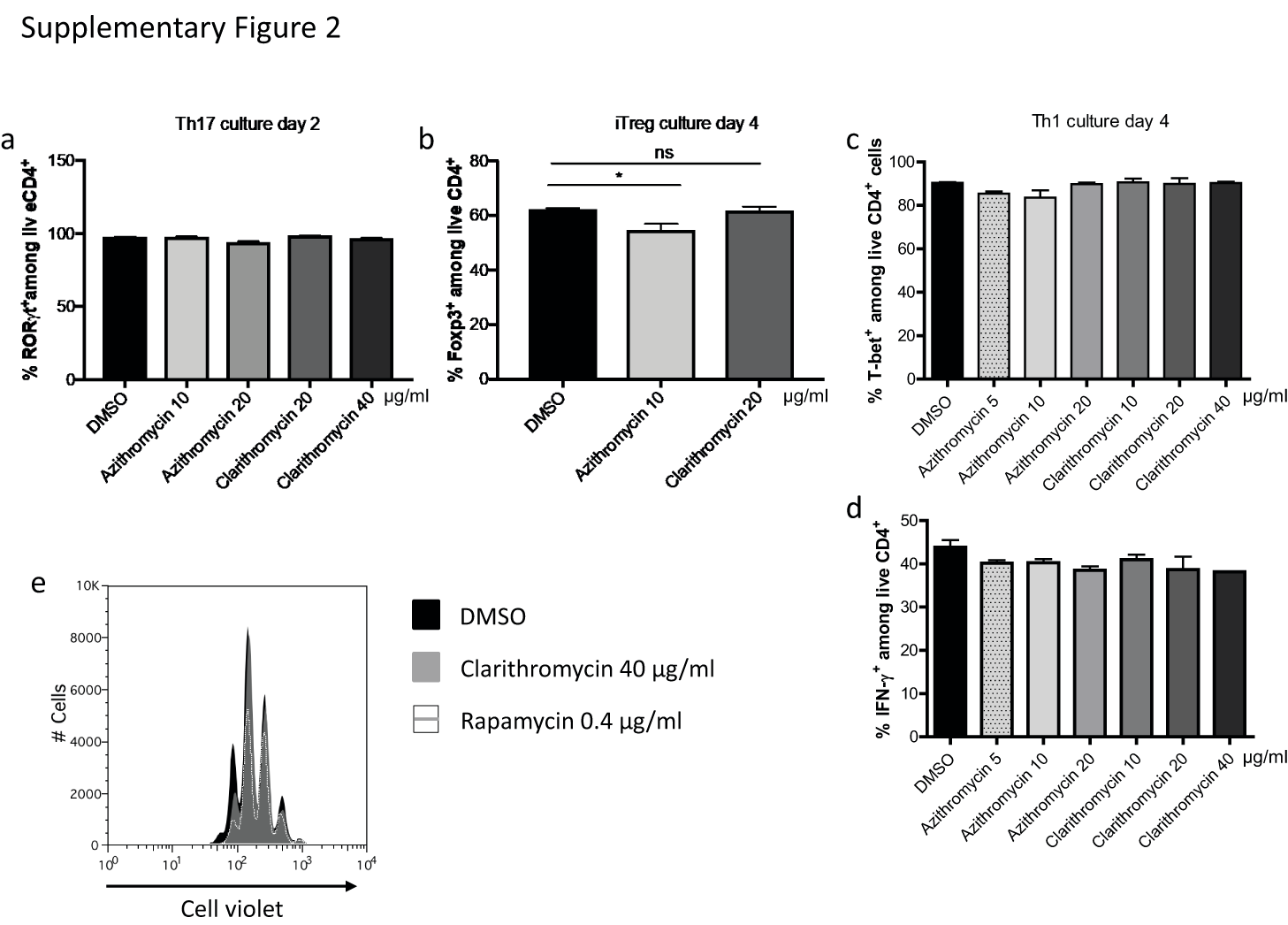

Supplement: Supplementary file 3 — In vitro effects of macrolide antibiotics on different Th subset differentiation and Th17 proliferation. a ROR gamma t expression in CD4+ T cells treated with the indicated macrolide concentrations at day 2 of Th17 culture. b Frequencies of FoxP3 expressing cells at day 4 of iTreg culture. c Frequencies of T-bet expressing cells and d IFN gamma producing CD4+ T cells at day 4 of Th1 culture. e Cell violet™ staining of Th17 differentiated CD4+ T cells at day 4 of culture. Data shown are representative of at least two individual experiments with three technical replicates each and depicted as mean ± S.E.M. Two-tailed, unpaired Student’s t test was used to determine significance between means of groups. ns (not significantly different); *p < 0.05 (PNG 4469 kb) [file 109_2021_2039_Fig7_ESM.png]

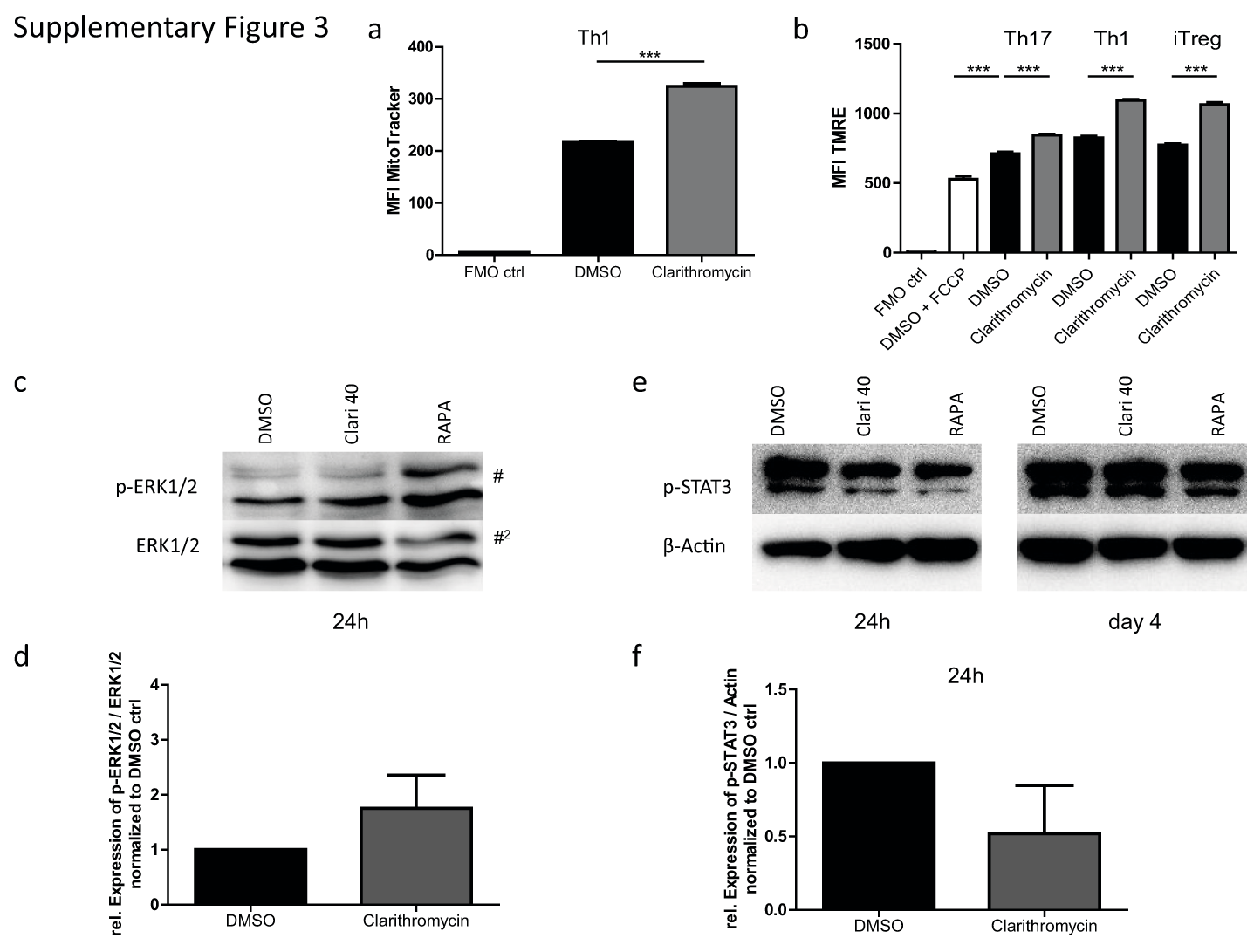

Supplement: Supplementary file 5 — In vitro effects of macrolide antibiotics on mitochondrial staining, ERK, and STAT3 phosphorylation. a Mitochondrial staining with MitoTracker Deep Red™ depicted as mean fluorescence intensity (MFI) at day 4 of Th17 culture. b Mitochondrial membrane potential-dependent staining with tetramethylrhodamine ethyl ester (TMRE) depicted as mean fluorescence intensity (MFI) at day 4 of Th17, Th1, and iTreg culture. Control cells were treated with FCCP to impair the mitochondrial membrane potential and thereby TMRE staining. Data shown are representative of three individual experiments with two technical replicates and depicted as mean ± S.E.M. Two-tailed, unpaired Student’s t test was used to determine significance between means of groups. ns (not significantly different); ***p < 0.0005. c Representative Western blots of p-ERK1/2 and ERK1/2 proteins of cells treated with 40 μg/ml Clarithromycin, 0.4 μg/ml Rapamycin, or DMSO as control and harvested at 24 h of Th17 culture. d Quantification of the expression of p-ERK1/2 to the total ERK1/2 expression normalized to this ratio in the DMSO control. e Representative Western blots of p-STAT3 and beta actin of cells treated with 40 μg/ml Clarithromycin, 0.4 μg/ml Rapamycin, or DMSO as control and harvested at 24 h or day 4 of Th17 culture. f Quantification of the expression of p-STAT3 to beta actin expression normalized to this ratio in the DMSO control at 24 h. Data shown are semi-quantitative analysis of two independent blots without further statistical analysis (PNG 4571 kb) [file 109_2021_2039_Fig8_ESM.png]

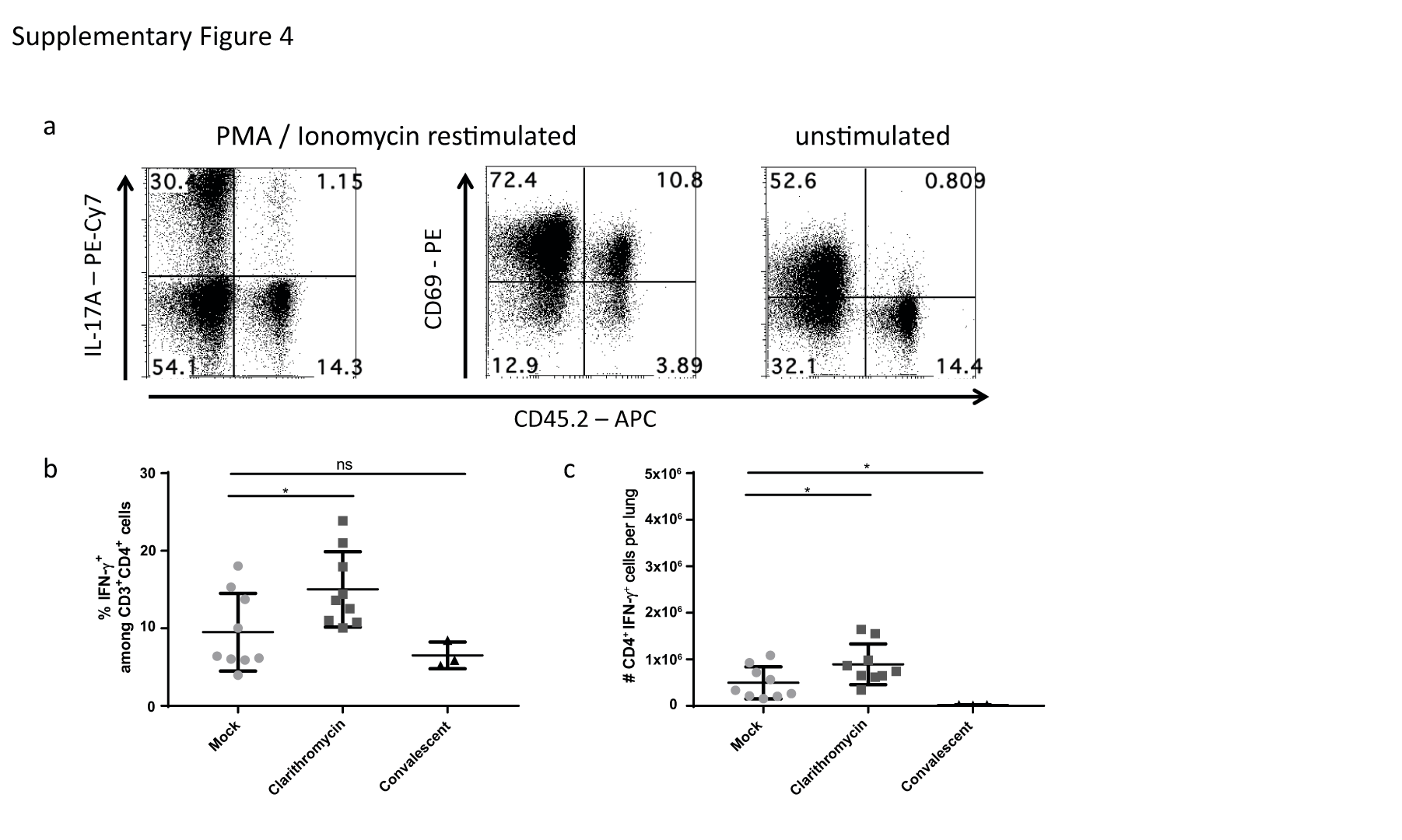

Supplement: Supplementary file 7 — Analysis of the memory Trm1 response and confirmation of tissue-residency of Trm17 cells. a Representative FACS plots of lung cells isolated from C57BL6 mice that have been infected with consecutive S. pneumoniae infections and intravenous application of a fluorescent conjugated antibody for the lymphocyte marker CD45.2 10 min before asphyxiation to discriminate blood circulating and lung resident cells. On the right-hand side, IL-17A staining showed only a negligible fraction of APC positive cells producing IL-17A, and on the left-hand side, CD69+ cells were not confounded by blood circulating cells, when stained directly ex vivo without restimulation. b Frequencies and c total cell numbers of IFN gamma producing CD4+ T cells at day 7 of the second S. pneumoniae infection. Data shown are two pooled independent experiments out of three (while the control group is only representative for one), and depicted as mean ± S.D. Two-tailed, unpaired Student’s t test was used to determine significance between means of groups. ns (not significantly different); *p < 0.05 (PNG 5706 kb) [file 109_2021_2039_Fig9_ESM.png]

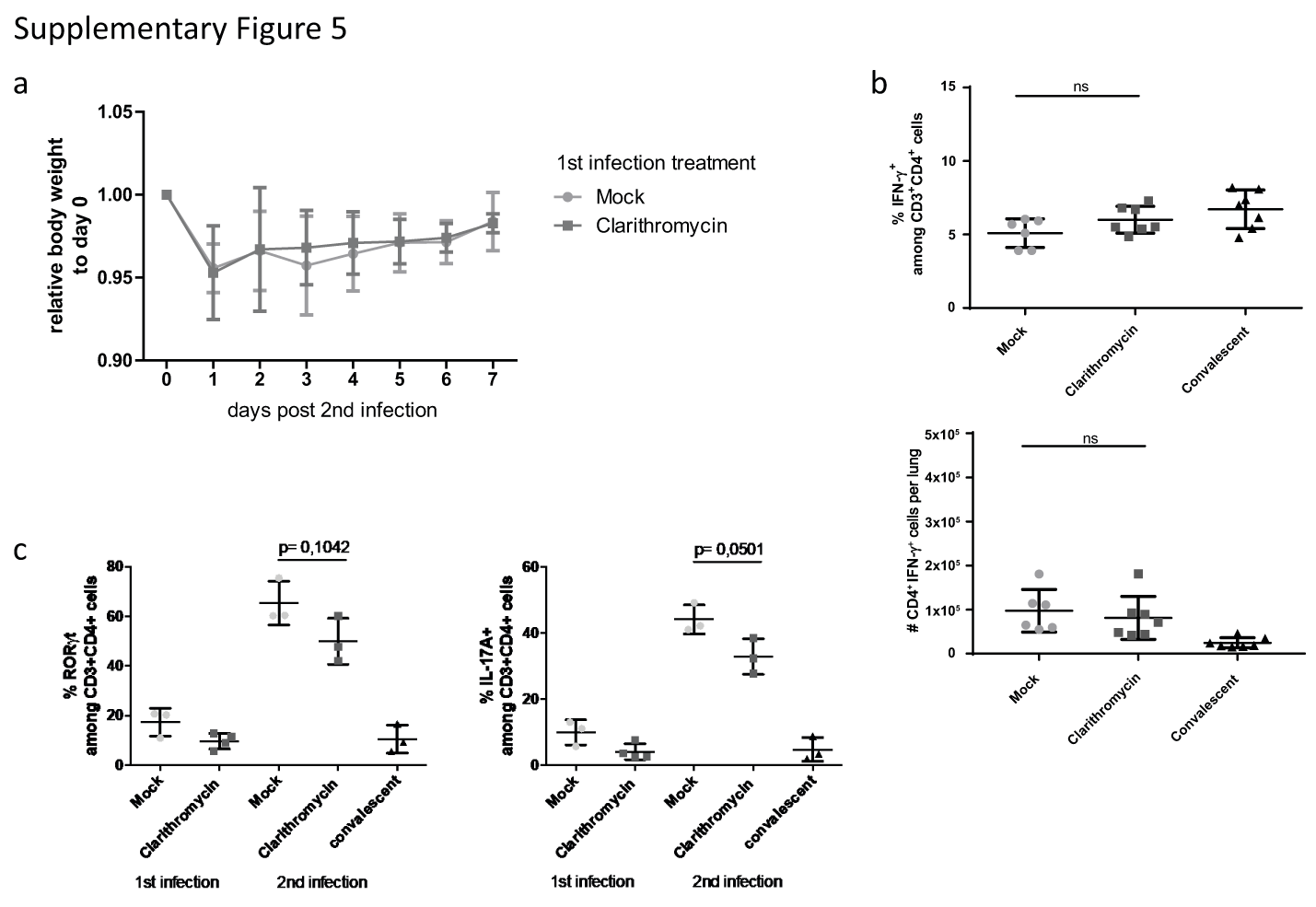

Supplement: Supplementary file 9 — Bodyweight curve of mice recovering from the second infection without Clarithromycin treatment; analysis of the memory Trm1 response and correlation of ROR gamma t expression to IL-17A production of Trm17 cells upon Clarithromycin treatment. a Bodyweight curve of mice infected for the second time with S. pneumoniae only treated with Clarithromycin or mock during the first infection. Data are pooled from three independent experiments. b Frequencies and total cell numbers of IFN gamma producing CD4+ T cells at day 7 of the second S. pneumoniae infection, while Clarithromycin treatment occurred during both infections. Data shown are two pooled independent experiments out of three, and depicted as mean ± S.D. c Frequencies of ROR gamma t expressing (left panel) and IL-17A producing CD4+ T cells from one experiment out of two, depicted as mean ± S.D. Two-tailed, unpaired Student’s t test was used to determine significance between means of groups. ns (not significantly different), p value as indicated (PNG 4128 kb) [file 109_2021_2039_Fig10_ESM.png]
